# The Extract of *Rhodobacter sphaeroides* Inhibits Melanogenesis through the MEK/ERK Signaling Pathway

**DOI:** 10.3390/md11061899

**Published:** 2013-06-03

**Authors:** Wen-Sheng Liu, Yu-Diao Kuan, Kuo-Hsun Chiu, Wei-Kuang Wang, Fu-Hsin Chang, Chen-Hsun Liu, Che-Hsin Lee

**Affiliations:** 1 Asia-Pacific Biotech Developing, Inc., Kaohsiung 806, Taiwan; E-Mails: wensheng5394@gmail.com (W.-S.L.); bio_apd@yahoo.com.tw (F.-H.C.); whoiamshin@gmail.com (C.-H.L.); 2 Graduate Institute of Basic Medical Science, School of Medicine, China Medical University, Taichung 404, Taiwan; E-Mail: yoite_to_miharu@hotmail.com; 3 Department and Graduate Institute of Aquaculture, National Kaohsiung Marine University, Kaohsiung 811, Taiwan; E-Mail: kuohsun@gmail.com; 4 Department of Environmental Engineering and Science, Feng Chia University, Taichung 407, Taiwan; E-Mail: wkwang@fcu.edu.tw; 5 Department of Biological Science and Technology, I-Shou University, Kaohsiung 840, Taiwan; 6 Department of Microbiology, School of Medicine, China Medical University, Taichung 404, Taiwan

**Keywords:** *Rhodobacter sphaeroides*, Lycogen™, melanogenesis

## Abstract

Reducing hyperpigmentation has been a big issue for years. Even though pigmentation is a normal mechanism protecting skin from UV-causing DNA damage and oxidative stress, it is still an aesthetic problem for many people. Bacteria can produce some compounds in response to their environment. These compounds are widely used in cosmetic and pharmaceutical applications. Some probiotics have immunomodulatory activities and modulate the symptoms of several diseases. Previously, we found that the extracts of *Rhodobacter sphaeroides* (Lycogen™) inhibited nitric oxide production and inducible nitric-oxide synthase expression in activated macrophages. In this study, we sought to investigate an anti-melanogenic signaling pathway in α-melanocyte stimulating hormone (α-MSH)-treated B16F10 melanoma cells and zebrafish. Treatment with Lycogen™ inhibited the cellular melanin contents and expression of melanogenesis-related protein, including microphthalmia-associated transcription factor (MITF) and tyrosinase in B16F10 cells. Moreover, Lycogen™ reduced phosphorylation of MEK/ERK without affecting phosphorylation of p38. Meanwhile, Lycogen™ decreased zebrafish melanin expression in a dose-dependent manner. These findings establish Lycogen™ as a new target in melanogenesis and suggest a mechanism of action through the ERK signaling pathway. Our results suggested that Lycogen™ may have potential cosmetic usage in the future.

## 1. Introduction

Melanin plays important photo-protective roles in the carcinogenic and deleterious effects of the ultraviolet radiation of solar light. Abnormal melanogenesis is a feature of many human skin diseases, including pigmentary disorders and melanoma. In Europe, skin lighteners are applied for the prevention and treatment of irregular hyperpigmentation, such as melasma and age spots. Past studies focused on the screening of nature compounds from plant sources for an anti-melanogenic agent [1,2]. In melanocytes, α-melanocytes stimulating hormone (α-MSH), through binding to the melanocortin-1 receptor (MC1R) and upregulation of the cAMP pathway, induces melanin synthesis. At least three enzymes are required for melanin synthesis, including tyrosinase, tyrosinase-related protein 1 (TRP1) and tyrosinase-related protein 2 (TRP2), through a transcriptional mechanism involving microphthalmia-associated transcription factor (MITF), the master gene of melanocyte differentiation. Some of the agents with antioxidative activity have been shown to play an important role in the inhibition of melanogenesis. Bacteria can produce some compounds in response to their environment. These compounds are widely used in cosmetic and pharmaceutical applications. Some probiotics have immunomodulatory activities and modulate the symptoms of several diseases. Previously, we found that the extracts of *Rhodobacter sphaeroides* (Lycogen™) inhibited nitric oxide production and inducible nitric-oxide synthase expression in activated macrophages [3]. Meanwhile, the effect of Lycogen™, a potent anti-inflammatory agent, was evaluated in mice with dextran sodium sulfate (DSS)-induced colitis. Oral administration of Lycogen™ reduced the expressions of proinflammatory cytokines (tumor necrosis factor-α and interleukin-1β) in mice [4]. In this study, we sought to investigate an anti-melanogenic signaling pathway in α-MSH-treated B16F10 melanoma cells and zebrafish.

## 2. Results

### 2.1. Evaluation of Anti-Melanogenic Activity of Lycogen™ *in vitro*

**Figure 1:**
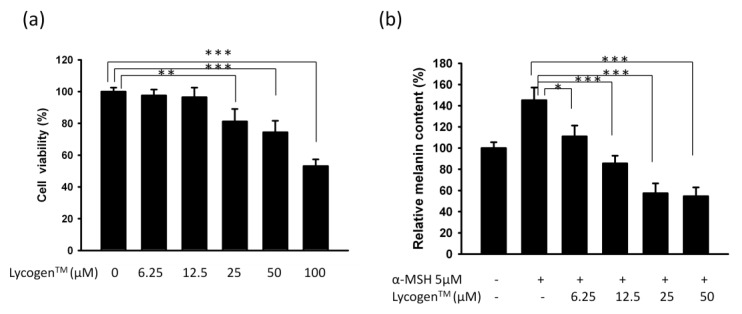
Effects of Lycogen™ on cell viability and melanin production in B16F10 cells. B16F10 cells were treated with indicated concentrations of Lycogen™ for 48h. (**a**) Cell viability was measured by WST-1 assay. (**b**) Effect of Lycogen™ on cellular melanin content. * *p* < 0.05; ** *p* < 0.01; *** *p* < 0.001. (Mean ± SD, *n* = 6). Each experiment was repeated three times with similar results.

In this study, we used mouse B16F10 melanoma cell to evaluate the anti-melanogenic activity of Lycogen™. The results for *in vitro* treatment of B16F10 cells with Lycogen™ for cell survival and melanin content are shown in Figure 1. Thus, doses (6.25 μM–12.5 μM) without significant cytotoxicity were chose to determine the effects of Lycogen™ on melanin production (Figure 1a). The melanin content of B16 cells increased considerably after stimulation by α-MSH. Treatment with Lycogen™ resulted in a significant and dose-dependent decrease in the melanin content of α-MSH-stimulated B16F10 cells (Figure 1b). Taken together, these results suggest that Lycogen™ influenced the melanogenesis in B16F10 cells.

### 2.2. Lycogen™ Dose-Dependently Downregulated the Expression Levels of Tyrosinase and MITF

**Figure 2:**
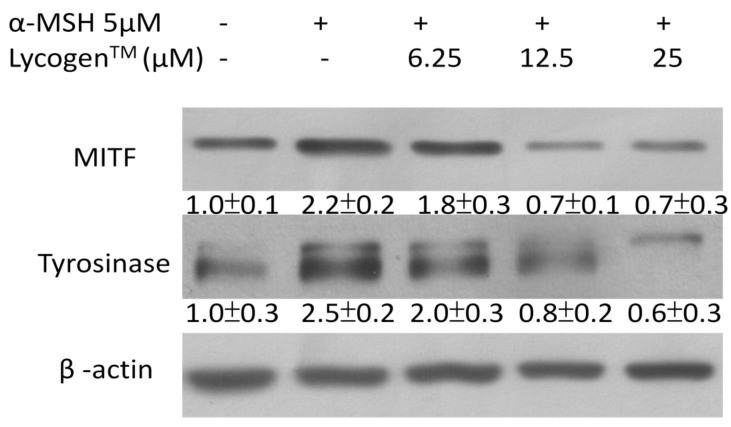
The expression levels of tyrosinase and microphthalmia-associated transcription factor (MITF) after Lycogen™ treatment. B16F10 cells were treated with Lycogen™ at the concentration of 6.25, 12.5 or 25 μM for 48 h. The protein expression was determined by immunoblotting. Inserted values indicated relative protein expression in comparison with β-actin. Each experiment was repeated three times with similar results.

To elucidate the mechanisms underlying the anti-melanogenic activity of Lycogen™, we first examined the expression levels of tyrosinase by immunoblotting. As shown in Figure 2, Lycogen™ dose-dependently reduced the expression of tyrosinase. Tyrosinases are transcriptionally regulated by MITF. Interestingly, we found that Lycogen™ dose-dependently inhibited MITF expression.

### 2.3. Effects of Lycogen™ on the Mitogen-Activated Protein Kinase (MAPKs) Signaling Pathway

**Figure 3:**
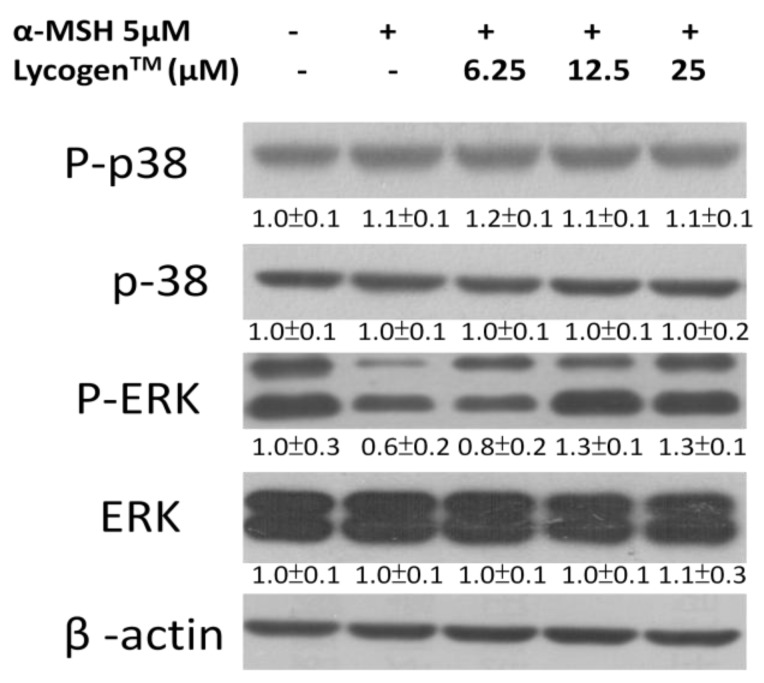
Effect of Lycogen™ on the expression levels of MAPKs and phosphorylated-MAPKs in cells. B16F10 cells were treated with Lycogen™ at the concentration of 6.25, 12.5 or 25 μM for 48h. The protein expression was determined by immunoblot analysis. The expression of β-actin served as the quantitative control. Inserted values indicated relative protein expression in comparison with β-actin. Each experiment was repeated three times with similar results.

**Figure 4:**
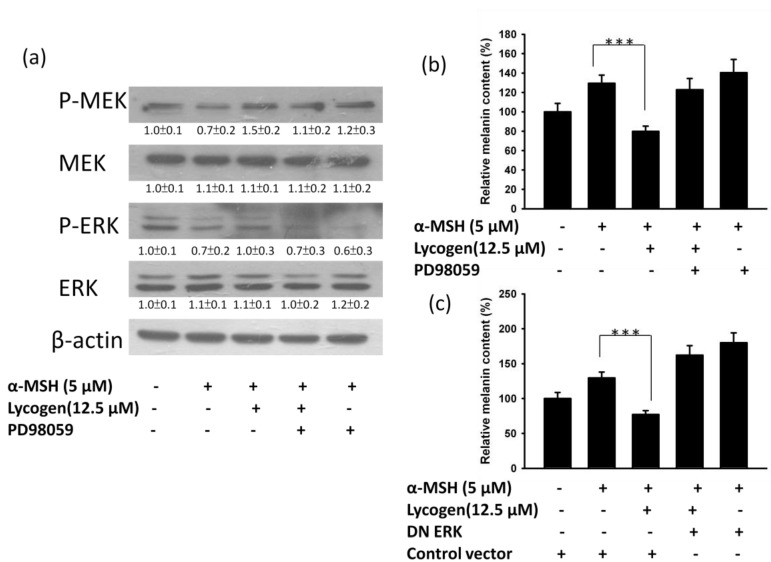
Lycogen reduces melanin content through the ERK pathway. B16F10 cells were treated with Lycogen™ at the concentration of 12.5 μM for 48h and PD98059 (50 μM) for 24h. (**a**) The protein and (**b**) melanin expression weremeasured. The expression of β-actin served as the quantitative control. Inserted values indicated relative protein expression in comparison with β-actin. (**c**) The melanin was detected in B16F10 cells transiently transfected withERK dominant negative or with control vector after treatment with Lycogen™.

**Figure 5:**
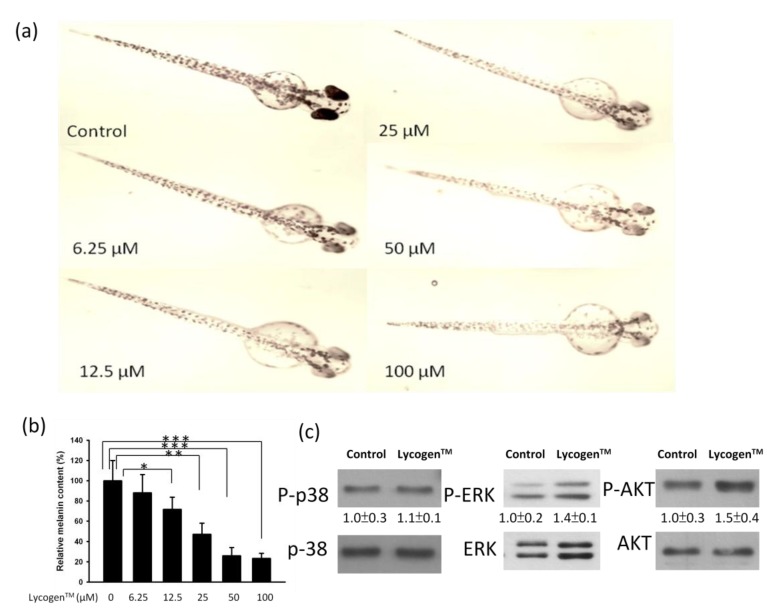
Effects of Lycogen™ on the pigmentation of zebrafish. (**a**) Image of zebrafish treated with Lycogen™. (**b**) Synchronized embryos were treated with various concentration Lycogen™ for 48h. Melanin pigment was determined by a photometric method. (Mean ± SD, *n* = 3). ** *p* < 0.01; *** *p* < 0.001. (**c**) The protein expression after Lycogen™ (12.5μM) treatment were measured by immunoblotting in zebrafish. The expression of total p38, ERK or AKT served as the quantitative control. Inserted values indicated relative protein expression in comparison with total protein. Each experiment was repeated three times with similar results.

MAPK kinase, including ERK and p38, plays an important role in melanogenesis [5,6]. Upregulation of ERK signaling is related to the downregulation of melanin synthesis. However, phosphorylation of p38 can upregulate the MITF expression. We examined the influence of Lycogen™ on the signaling pathway of p38 and ERK in an attempt to further understand the molecular mechanism involved in the anti-melanogenic activity of Lycogen™ by immunoblotting. In this study, we found that α-MSH significantly reduced the ERK signaling pathway (Figure 3). However, Lycogen™ reversed the phenomenon. Lycogen™ dramatically increased the ERK signaling pathway. Meanwhile, p38 signaling pathway was not influenced by Lycogen™. Furthermore, the α-MSH-induced response of MAPK/ERK pathway associated factors, ERK and MEK, were determined (Figure 4a). However, MEK and ERK phosphorylation significantly decreased after α-MSH treatment. In order to investigate the relationship between melanogenesis and the ERK pathway, B16F10 cells were treated with inhibitor PD98059 before Lycogen™ treatment. PD98059 is a potent and selective inhibitor of MAP kinase kinase (also known as MAPK/ERK kinase or MEK kinase). It mediates its inhibitory properties by binding to the ERK-specific MAP kinase MEK, therefore preventing phosphorylation of ERK1/2 (p44/p42 MAPK) by MEK. Immunoblot analysis showed that PD98059 reduced the expression of ERK phosphorylation. The content of melanin also dramatically increased after PD98059 treatment (Figure 5b). Lycogen™ did not reverse melanogenesis by PD98059. The similar results were also observed in B16F10 cells after ERK dominant negative plasmids transduction (Figure 4c). Consistent with PD98059 treatment, ERK dominant negative plasmid abolished the inhibitory effect of Lycogen™ on melanin synthesis. The ERK signaling inhibitors blocked the hypopigmenting effects induced by Lycogen™. These results suggested that Lycogen™ increased MEK/ERK cascade, promoting MITF degradation.

### 2.4. Evaluation of Anti-Melanogenic Activity of Lycogen™ *in vitro*

Zebrafish have been established as a new *in vivo* model for evaluating the depigmenting activity of melanogenic regulatory compounds. No significant toxicity of Lycogen™ was observed in zebrafish embryos at the tested concentrations up to 100 μM (Figure 5a). However, treatment of the embryos with Lycogen™ during 48 h significantly reduced the skin melanin in the developed larvae (Figure 5a). The melanin content of Lycogen™-treated zebrafish was dose-dependently decreased compared to that of untreated fish (Figure 5b). To understand the postulated signal mechanism involved in the inhibitory effect of Lycogen™ on melanin synthesis in zebrafish, zebrafish embryos were treated with 12.5 μM for 48 h. The protein expression of p38, ERK and AKT were determined by Western blotting. The treatment of Lycogen™ led to a slight increase in phosphorylated-ERK and phosphorylated-AKT. Consistent with B16F10 cells, the expression of phosphorylated-p38 was not influenced after Lycogen™ treatment. The results point out that Lycogen™ inhibited the melanogenesis *in vivo*.

## 3. Discussion

We had found that Lycogen™ could reduce the melanin production *in vitro* and *in vivo*. Although B16F10 cells expressed MITF, it was reduced after treatment with Lycogen™, as assessed by Western blot analysis, in a dose-dependent manner. Herein, we want to point out that Lycogen™ could inhibit melanin production by targeting the ERK signaling pathway. Lycogen™ is able to serve as a potential melanogenesis inhibitor. The suppressed activation of cAMP response element-binding protein (CREB) via the downregulation of p38 had been reported in hypopigmentation [6]. Herein, we found that Lycogen™ did not influence the phosphorylation of p38. It was reported that the activation of ERK resulted in the phosphorylation of MITF at serine 73, which induced the subsequent ubiquitin-dependent proteasomal degradation [7]. Meanwhile, in the present study, the involvement of the ERK signaling pathway was confirmed by the treatment of PD98059 or the ERK dominant negative plasmid. A similar anti-melanogenic effect was also described in that c-phycocyanin raised the level of ERK phosphorylation to inhibit the biosynthesis of melanin [7]. These observations might suggest that the constitutes of Lycogen™ could function as an ERK activator to regulate melanin synthesis. These results showed that ERK phosphorylation is significantly upregulated when B16F10 cells and zebrafish are treated with Lycogen™, which suggests that Lycogen™ induces hypopigmentation by increasing ERK phosphorylation *in vitro* and *in vivo*. The activation of AKT is responsible for suppression of melanin synthesis in melanoma cells, and specific inhibition of the AKT pathway stimulates melanin synthesis. In the present study, zebrafish treated with Lycogen™ also increased the expression of phosphorylated-AKT. Thus, our results indicate that Lycogen™-induced melanin reduction may be mediated by the ERK and AKT signaling pathway. Lycogen™ contains ζ-carotene, neurosporene, spheroidenone and methoxyneurosporene, according to nuclear magnetic resonance spectroscopy analysis. ζ-Carotene is the precursor of neurosporene, which, in turn, is the precursor of lycopene [8]. Lycopene, as an antioxidative agent, prevents the production of melanin [9]. Meanwhile, neurosporene itself has the ability to protect irradiation with UV-B [10]. Lycogen™ inhibited NO production and iNOS expression in activated macrophages [3] and was capable of improving colonic damage in DSS-induced colitis [4]. Further work is warranted to elucidate the active ingredient(s) in Lycogen™ for hypopigmentation. These findings point out that Lycogen™ might contribute to its therapeutic effect on anti-melanogenesis.

## 4. Experimental Section

### 4.1. Lycogen™, Cells, Mice and Zebrafish

*R.* *sphaeroides* (WL-APD911) was isolated from mutants using chemical mutagenesis (Bioresource Collection and Research (BCRC), Hsinchu, Taiwan). The *R. sphaeroides* was cultured in broth. After harvesting, the bacterial broth was centrifuged and washed with ethanol. The bacterial residue is extracted with acetone and then centrifuged by 7500 rpm for 5 min. The supernatant is filtered through filter paper and a 0.2 μm filter into a round-bottomed flask. The color of the final supernatant is dark red. Acetone is removed completely in an oven at 55 °C. The extract of *R. sphaeroides* was named Lycogen™. Lycogen™ are available from Asia-Pacific Biotech Developing, Inc. (Kaohsiung, Taiwan). Murine B16F10 cells are cultured in Dulbecco’s modified Eagle’s medium containing 10% fetal bovine serum, 1% glutamine and 50 μg/mL gentamicin at 37 °C in 5% CO_2_ [11,12]. Adult zebrafish were obtained from a commercial dealer, and 10–15 fishes were kept in a 5 L acrylic tank with the following conditions; 28.5 °C, with a 14/10 h light/dark cycle. Zebrafish were fed three times a day, 6 days/week, with flake food supplemented with live brine shrimp (*Artemia salina*). Embryos were obtained from natural spawning that is induced at the morning by turning on the light. Collection of embryos were completed within 30 min. The experimental protocol adhered to the rules of the Animal Protection Act of Taiwan and was approved by the Laboratory Animal Care and Use Committee of the China Medical University.

### 4.2. Determinations of Anti-Melanogenic Activity in Zebrafish

Synchronized embryos were collected and arrayed by pipette, three to four embryos per well, in 96-well plates containing embryo medium. Lycogen™ were dissolved in water, then added to the embryo medium for 48 h. The effects on the pigmentation of zebrafish were observed under the stereomicroscope. For observation, embryos were dechorionated by forceps, anesthetized in tricaine methanesulfonate solution (Sigma-Aldrich, St. Louis, MO, USA), mounted in 3% methyl cellulose on a depression slide and photographed under the stereomicroscope MZ16 (Leica Microsystems, Ernst-Leitz-Strasse, Germany). The signals were quantified with ImageJ software [13].

### 4.3. Assay of Cell Proliferation

Cells (10^5^/well) were treated with various concentration of Lycogen™ in culture medium for 48 h. The medium was removed, washed and replenished with fresh medium supplemented with 2% FBS. Cell proliferation was assessed by the colorimetric WST-1 assay (Dojindo Labs, Tokyo, Japan), according to the manufacturer’s instructions [14].

### 4.4. Determination of Melanin Content

At the end of the cell culture, the cells were harvested and washed twice with PBS. The pelleted cells were lysed in cold lysis buffer (20 mM sodium phosphate pH 6.8, 1% Triton X-100, 1 mM PMSF and 1 mM EDTA). After centrifugation at 15,000× *g* for 15 min, the melanin pellets were dissolved in Soluene-350 (Perkin-Elmer, Waltham, MA, USA) for 15 min at 100 °C. The absorbance at 500 nm was measured [15]. The protein content in each sample was determined by bicinchoninic acid (BCA) protein assay (Pierce Biotechnology, Rockford, IL, USA). The working concentrations were 20 μM PD98059 (Sigma-Aldrich). Cells were pretreated inhibitors for 1 h; then, Lycogen™ was added to cells. Lycogen™-treated or untreated cells were lysed in cold lysis buffer (20 mM sodium phosphate pH 6.8, 1% Triton X-100 and 1 mM PMSF).

### 4.5. Western Blot Analysis

To inhibit the ERK signal pathway, cells were pretreated PD98059 for 1 h; then, Lycogen™ was added to cells for 48 h. The B16F10 cells were transfected with ERK dominant negative plasmids by Lipofectamine 2000 (Invitrogen, Carlsbad, CA, USA). The ERK dominant negative mutant was a gift from Dr. M. Cobb (South-Western Medical Center, Dallas, TX, USA). Cell lysates were prepared by extracting proteins with lysis buffer. Proteins from total cell extracts were fractionated on SDS-PAGE, transferred onto Hybond-enhanced chemiluminescence nitrocellulose membranes (Amersham, Little Chalfont, UK) and probed with primary antibodies against tyrosinase (Santa Cruz Biotechnology, Santa Cruz, CA, USA), MITF (Santa Cruz Biotechnology), ERK (Abcam, Cambridge, UK), phosphor-ERK (Abcam), AKT (Cell Signaling, Danvers, MA, USA), phosphorylated-AKT (Cell Signaling), p38 (Cell Signaling), phosphorylated-p38 (Cell Signaling) or monoclonal antibodies against β-actin (AC-15, Sigma Aldrich). Horseradish peroxidase-conjugated secondary antibodies were used, and protein-antibody complexes were visualized by an enhanced chemiluminescence system (Amersham) [16]. The signals were quantified with ImageJ software [13].

### 4.6. Statistical Analysis

All data were expressed as the mean ± standard deviation (SD). The one-way ANOVA test was used to determine differences between groups. Any *p*-value less than 0.05 is considered statistically significant.

## 5. Conclusions

In conclusion, our work has identified Lycogen™ as an anti-melanogenic agent with the capacity to ameliorate α-MSH induced-hyperpigmentation. However, we also elucidated the underlying mechanism of the therapeutic effects of Lycogen™ therapy in anti-melanogenesis.
